# Revealing new depths of information with indentation mapping of microstructures

**DOI:** 10.1557/s43577-025-00919-6

**Published:** 2025-06-04

**Authors:** Edoardo Rossi, Christophe Tromas, Zhiying Liu, Yu Zou, Jeffrey M. Wheeler

**Affiliations:** 1 Department of Civil, Computer Science and Aeronautical Technologies Engineering, Università degli Studi Roma Tre, Rome, Italy; 2 Institut Pprime, Université de Poitiers/UPR 3346 CNRS/ISAE-ENSMA, Poitiers, France; 3 Department of Mechanical Engineering, City University of Hong Kong, Kowloon, Hong Kong; 4 Department of Materials Science and Engineering, University of Toronto, Toronto, ON Canada; 5 Laboratory for Nanometallurgy, Department of Materials, ETH Zürich, Zurich, Switzerland

**Keywords:** Nanoindentation, Hardness, Scanning electron microscopy (SEM), Machine learning, Microstructure, Statistics/statistical methods

## Abstract

Nanoindentation is crucial in materials science for assessing mechanical properties in submicrometer volumes, and high-speed nanoindentation mapping has evolved it from a localized measurement technique into a scanning-probe-like approach for microstructures, delivering large-area, high-resolution mechanical property maps with more than 200,000 indents in hours. Such mapping enables direct imaging of hardness and modulus variations, phase boundaries, and local deformation behaviors in materials where heterogeneity governs mechanical performance. By correlating these mechanical maps with composition, orientation, and phase data from complementary analytical techniques, deep multidimensional data sets reveal the complex interplay between structure, processing, and properties. Such data sets increasingly demand advanced statistical clustering, machine learning, and deep learning for classification, trend extraction, and phase identification. Moving forward, high-speed nanoindentation is anticipated to operate under *operando* conditions and advanced mechanical modalities, offering new insights into interfacial deformation, anisotropic behavior, and the broader challenges of materials design and performance.

**Graphical abstract:**

Schematic representation of high-speed nanoindentation mapping revealing microstructural heterogeneities in mechanical response. The indenter tip rapidly probes the surface, generating property maps sensitive to features such as twinning, recrystallization, segregation, precipitates, and sintered phases. These mechanical maps can be directly correlated with crystallographic and phase information from Electron Backscatter Diffraction (EBSD) and elemental composition from Energy-Dispersive X-ray Spectroscopy (EDS). Measurements can be performed operando, i.e., under real-time and service-relevant environmental conditions (e.g., temperature, atmosphere), enabling direct analysis of structure–property–performance relationships at the microstructural scale.
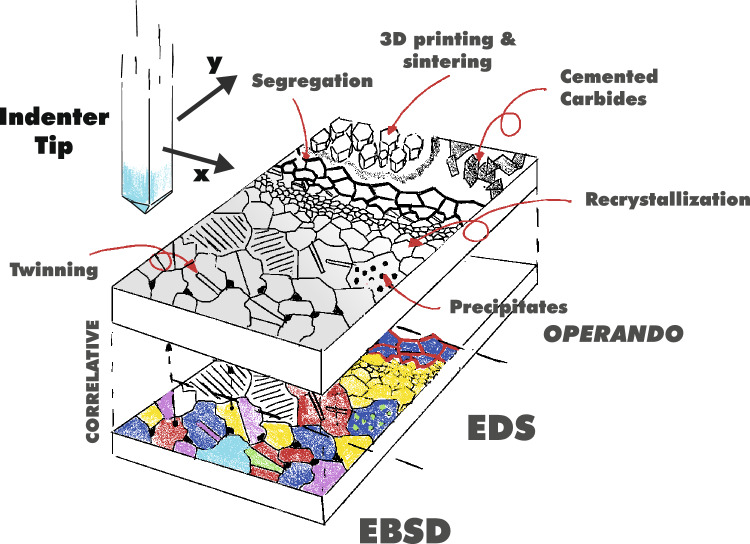

## Introduction

Nanoindentation emerged in the early 1990s through advancements in instrumented indentation testing and analysis models, enabling submicron material characterization.^1–3^ The ability to perform indentation tests with penetration depths as shallow as 50 nm, enabled by a typical Berkovich tip radius of ~50 nm, established nanoindentation as an essential technique, measuring the mechanical properties of thin films for the first time. Advancements such as automated indentation arrays expanded its use to heterogeneous materials, enabling phase identification and property mapping.^4–8^

What makes mechanical mapping appealing from a scientific perspective? A map transforms raw data into a visual format that enhances understanding. The visual cortex processes images holistically, while the text is analyzed word by word. Maps offer immediate context, making them accessible to a broader audience with less technical expertise. They help identify microstructural features, patterns, and anomalies that could be missed in raw data and enable comparisons across different regions, particularly in visualizing the relationship between mechanical properties and microstructure, such as crystallographic orientation or chemical composition.

**Figure 1:**
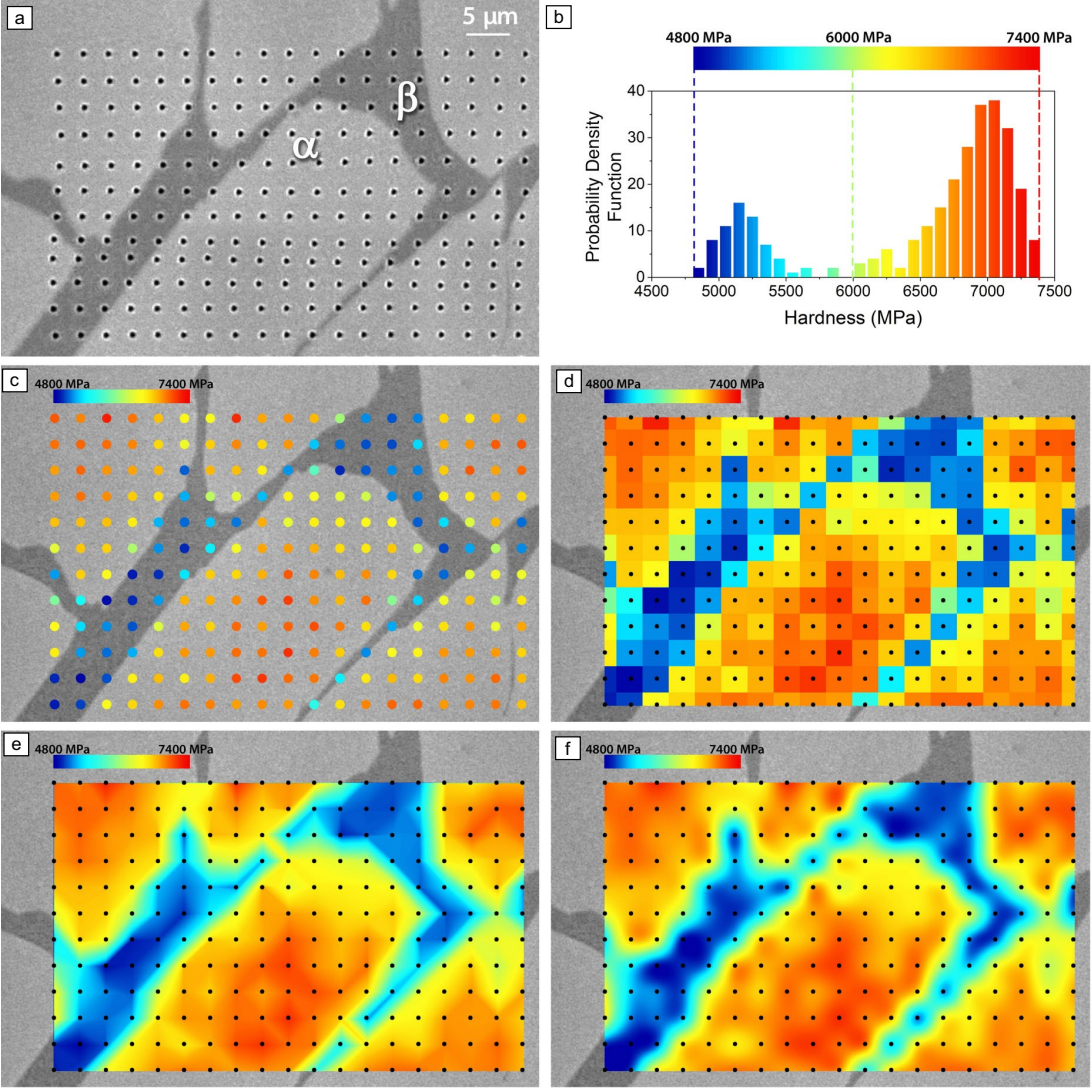
Nanoindentation mapping of hardness in an α + β titanium alloy. (a) Optical microscopy image of the indentation matrix, (b) hardness statistic and color scale, (c) discrete data mapping, (d) nearest-neighbor interpolation, (e) bilinear interpolation, (f) bicubic interpolation. The black dots represent the position of the nanoindentation measurements.

However, nanoindentation grids provide intrinsically discrete data. Even if the latest generation of nanoindenters can perform thousands of indentations with a step size of less than a micrometer, there will always be gaps between indents that cannot be probed. This is no longer due to positioning accuracy but instead to the spacing-to-depth ratio. In optimal conditions, such as with a brand-new Berkovich tip (radius ≤20 nm) and well-prepared surfaces, depths down to 15 nm can be achieved, even if, at these depths, indentation size effects (ISEs) and non-self-similarity influence accuracy. This results in an indent diameter of a few hundred nanometers, with the surrounding plastic zone often exceeding the residual indent: step size between the indents must ensure no interactions between the nearest neighboring ones.^9^ Filling this gap between measurement points requires interpolation methods. This is a double-edged sword: while it produces a smoother map, making it easier to identify specific regions, it can also mask smaller intermediate regions or local fluctuations.** Figure** 1 illustrates the different interpolation methods in the case of a nanoindentation hardness map obtained on an α + β titanium alloy. The beta phase is organized as thin lamellas with dark contrast on the optical microscopy image (Figure 1a). The indents have been performed in displacement control with a penetration depth of 100 nm. The step size has been chosen to be 3 µm, a relatively large value to illustrate the point.

Figure 1b illustrates how the statistics of the measured values aid in selecting color scales to enhance phase distinction. However, improper scaling can distort data interpretation by masking variability or blurring trends, potentially obscuring key microstructural features. Nearest-neighbor interpolation, the simplest approach, assigns each pixel the value of the closest indent (Figure 1d), preserving discrete measurements but potentially oversimplifying gradients. The bilinear interpolation (Figure 1e) calculates intermediate values by linear interpolation between four surrounding measurement points (2 × 2), while the bicubic interpolation (Figure 1f) achieves smoother rendering by applying cubic spline interpolation across 16 points (4 × 4) surrounding the pixel under consideration. Although they produce different visual outputs, neither method is inherently more precise.

Figure 1 illustrates both the benefits and the risks of nanoindentation mapping. While in Figure 1, the alpha and beta phases are clearly identified, enabling pattern recognition of mechanical contrasts, simple statistics alone would not allow for effective phase deconvolution.^8^ However, fine beta lamellae could be missed due to grid resolution, creating a misleading perception of precision. In such cases, visualizing discrete raw data (Figure 1c) helps mitigate interpolation-induced biases.

To effectively address this issue, it is crucial to carefully select the indentation spacing or resolution of an indentation map to capture the relevant microstructural features accurately. This can be achieved by creating smaller, initial maps across a range of resolutions to ensure that the features of interest are clearly imaged. Although nanoindentation maps require careful presentation, they provide accessible, high-density data visualization, elevating the technique to **mechanical microscopy**. Acknowledging this, instrument manufacturers now integrate high-performance mechanical mapping as a standard feature in modern nanoindentation systems.

## State of the art

Advancements in nanoindentation hardware have markedly improved speed, data acquisition, and precision, enabling high-speed nanoindentation mapping with submicron resolution.^10,11^

Modern systems can perform individual indents in less than 1 s, generating high-density maps with hundreds of thousands of indents covering areas from square microns to square millimeters.

The addition of continuous stiffness measurement (CSM) transforms the data set into a multidimensional tensor,^3^ linking each (*x*, *y*) coordinate with a comprehensive load-depth signal. CSM captures additional time- or depth-dependent mechanical properties, such as stiffness and modulus evolution.

However, high-speed operation introduces strain-rate effects—penetrating 50 nm in 1 s results in a strain rate of ~1/s, potentially reaching 10/s in faster processes.

These exceptional performances are achieved through the interplay of three key frequencies: actuation frequency, read-out frequency, and CSM oscillation frequency. The actuation frequency governs the control of loading and displacement and is influenced by system time constants, with advancements in feedback systems and phase-lock amplifiers reducing response delays. The read-out frequency, often higher than the actuation frequency for optimal sampling, determines the system’s data acquisition rate, reaching up to 1–5 MHz in modern instruments. Higher CSM oscillation frequencies can also exacerbate plasticity-related errors,^12–14^ especially in materials with high elastic-to-plastic ratios. These errors arise from nonelastic deformations induced by sinusoidal CSM oscillations, distorting stiffness measurements, and complicating modulus evaluations. Proper tuning of actuation, readout, and CSM frequencies is, therefore, critical to optimizing measurement accuracy and minimizing distortions in modulus evaluations.

Nanoindentation tests can be terminated based on target load or displacement. Displacement-targeted tests are ideal for materials with property gradients, while load-targeted tests offer precise force control for strain-rate sensitivity studies. In load-controlled systems, proportional integral derivative (PID) controllers maintain stable displacement, while displacement-controlled systems achieve this intrinsically. At high actuation frequencies, direct displacement control could provide better precision in maintaining indentation depth.

## Exploring local microstructure–property relationships in emerging materials using nanoindentation mapping

Nanoindentation mapping is providing new depths of information in a wide range of advanced materials with a diverse spectrum of microstructures, where detailed insights into gradients, interfaces, and microstructural variations are nontrivial, including additively manufactured alloys, hard and refractory metals, high-entropy alloys (HEAs), cementitious materials, advanced coatings (e.g., thermal barrier or cladding^11,15^), ceramics, polymer composites, and natural materials, where nanoindentation mapping has been shown to provide unique insight into their structure–processing–properties relationships, playing a critical role in various high-demand industries, such as civil construction, aerospace, automotive, energy, and biomedical engineering.

**Figure 2:**
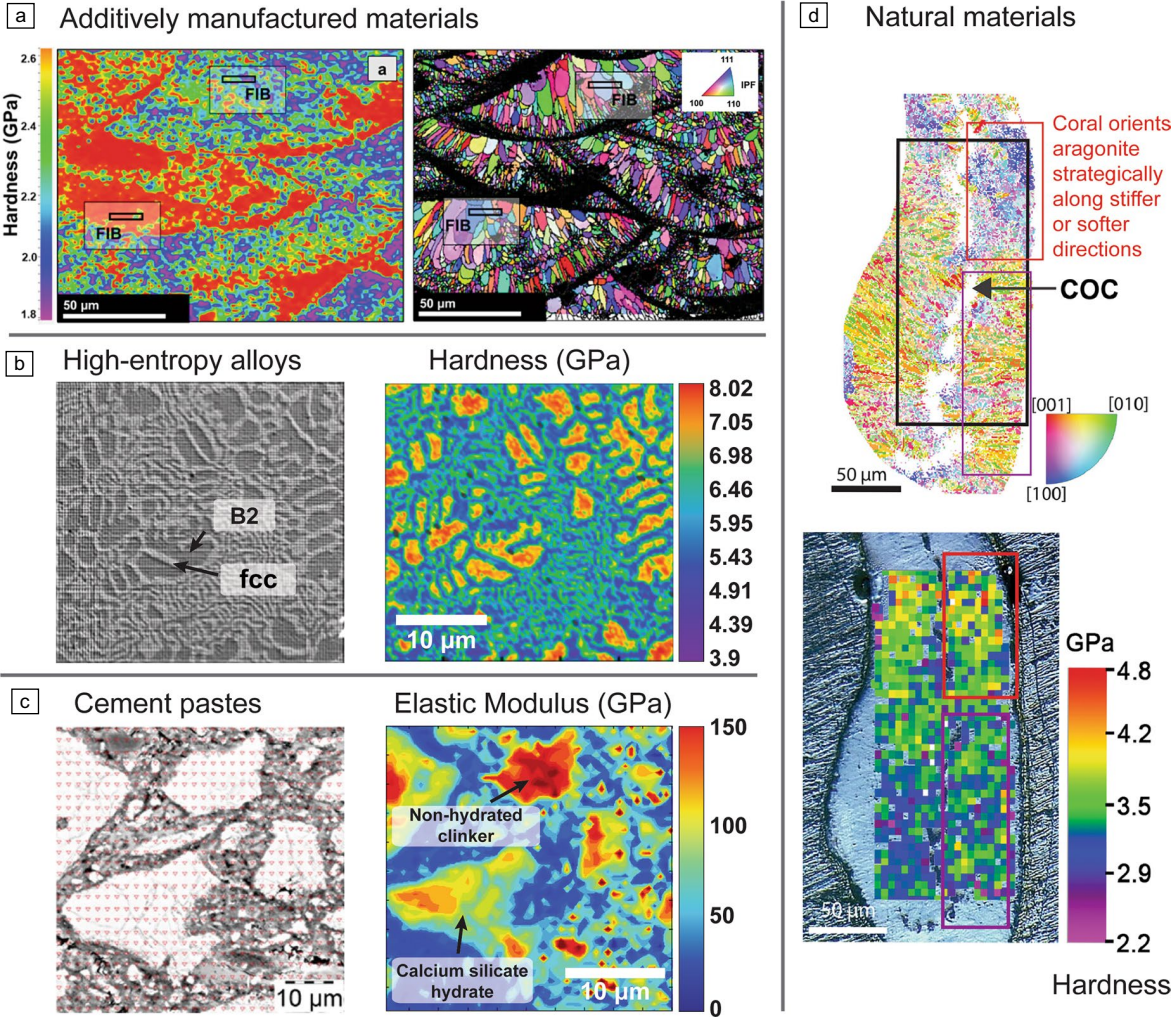
Nanoindentation mapping provides localized microstructure–property relationships in a wide range of materials: (a) additively manufactured materials (Reprinted with permission from Dhal et al.^16^ © 2024 Elsevier). FIB, focused ion beam; IPF, inverse pole figure. (b) High-entropy alloys (reprinted with permission from Wang et al.^17^ © 2023 Elsevier). B2, ordered body-centered cubic. (c) Cement pastes (reprinted with permission from Sebastiani et al.^18^ © 2016 Elsevier), and (d) natural materials (reprinted with permission from Moynihan et al.^19^ © 2022 Elsevier). COC, center of calcification.

**In additive manufacturing (AM)**, metallic materials often exhibit heterogeneous microstructures (**Figure** 2a), which can impact their performance and reliability. Quantifying the hardness variation between the interior and boundary of the melt pools for laser-based AM,^20^ examining coherent precipitation during cyclic remelting and reheating, local heterogeneities in multiphase alloys (e.g., hardness and modulus of alpha and beta phases inside molten pools in AM-produced Ti–6Al–2Zr–Mo–V),^21,22^ also identifying phase segregations (mechanical profile of the alpha/beta boundaries),^23,24^ or filament extrusion-based additive manufacturing and solid-state AM processes,^25,26^ or localized deformation in melt-pool boundaries in AM-produced aluminum alloys^20^ are just some of the potential applications in the field.

**Composite materials** inherently challenge conventional mechanical testing due to their complex structure—distinct phases of matrix and reinforcement interact at multiple length scales to produce enhanced mechanical properties—and mapping provides a means to optimize their performances by bridging microstructure with localized mechanical response. Mechanical microscopy offers a unique ability to spatially resolve these interactions to understand stress distributions, load transfer, and failure mechanisms. Examples are polymer-matrix composites, where mapping the interaction between reinforcement phases and binders provides insights into their deformation behavior,^27^ and harder systems, such as SiC-carbon fiber composites^28^ or polycrystalline cubic boron nitride, or tungsten carbide,^29^ where variations in hardness and elastic modulus at interfaces directly influence wear/erosion resistance and toughness.^17^

High-speed nanoindentation is a vital tool for linking chemical composition with mechanical properties in **HEAs**. The complexity of multiple elements with varying melting points causes significant mechanical variations at the micro- and nanoscales, and examples such as these of Kalali et al.^30^ and Wang et al.^17^ (Figure 2b) demonstrate how segregation at boundaries could be studied and linked with local mechanics. A key trend is the development of HEA deposition techniques over large surfaces with graded variations in composition, enabling combinatorial alloy libraries, as will be discussed later.^31^

**Cement pastes** have historically been a focus for nanoindentation mapping techniques to resolve their hierarchical microstructures**.** Traditional methods, such as those by Constantinides and Ulm,^32,33^ used low-throughput grids and probabilistic deconvolution to classify silicate hydrates, but sparse data limited the accurate mapping of spatial gradients and phase transitions. High-speed mapping changed this paradigm, allowing insights such as (1) direct visualization of interfacial transition zones (ITZs), which are crucial for understanding stress transfer and cracking;^34^ (2) quantification of local transformations during hydration and degradation, something earlier methods could only approximate through indirect modeling;^35^ and (3) phase development over time (i.e., mechanical responses in the early stages of hydration; see map in Figure 2c).^18^

High-speed nanoindentation has also been applied to **natural materials**^36,37^ revealing microscale mechanical gradients, and their structural correlations. Xu et al.^38^ investigated wood’s cellular heterostructures to inform biomass processing and lightweight structural design, while Moynihan et al.^19^ demonstrate how nanoindentation mapping reveals mechanical anisotropy in coral skeletons by correlating Young’s modulus and hardness with crystal orientation and hierarchical architecture. This approach provides unique insights into biomineralization and structural resilience (Figure 2d).

These advanced applications and the novel insights provided by high-speed mapping underscore its indispensability for materials where heterogeneity governs performance, enabling a deeper understanding of gradients, interfaces, and phase interactions across diverse fields.

## Correlative mechanical microscopy

One of the greatest potential applications of high-speed nanoindentation mapping is in combination with other analytical techniques. By correlating mechanical property maps with these techniques, the complex relationships between structure, processing, composition, and mechanical properties can be directly determined at the local microstructural level. The fine length scale of nanoindentation mapping allows for correlation with a wide variety of analytical characterization techniques: energy- or wavelength-dispersive x-ray spectroscopy (EDS/WDS) for composition, electron backscatter diffraction (EBSD) for crystallographic structure, Raman spectroscopy for bonding states, laser-based time-domain thermoreflectance (TDTR) for thermal properties,^39^ and many more.

**Figure 3:**
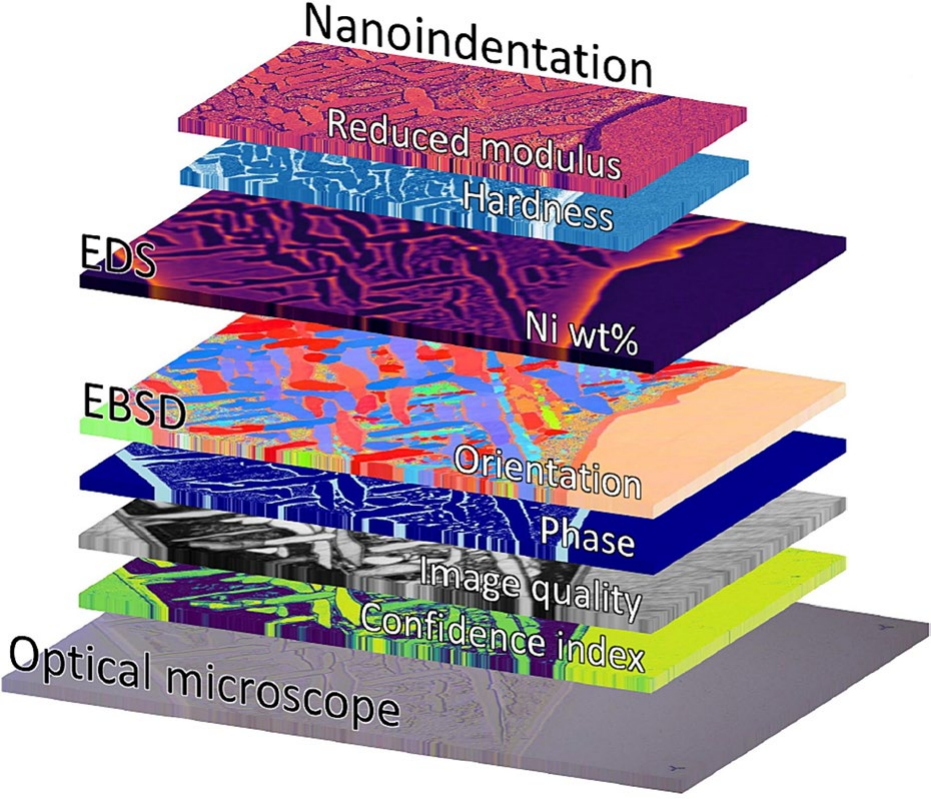
Illustration of a multimodal, correlated data set of nanoindentation, energy-dispersive x-ray spectroscopy (EDS), electron backscatter diffraction (EBSD), and optical microscopy performed on a fragment of the Seymchan meteorite.^41^ Reprinted with permission from Seehaus et al.^41^ © 2023 Elsevier.

At the outset, a clear distinction should be made between **visual comparison**, where maps are manually aligned and presented side-by-side, and **correlated analysis** with mutually registered data sets with numerical spatial alignment—as illustrated by** Figure** 3. Only the latter enables statistical and multidimensional analysis of microstructure properties (e.g., hardness based on composition and orientation). Data correlation and numerical alignment use domain transformation methods such as translations, rotations, and scaling to accurately align data sets, which can be challenging due to varying resolutions, sampled volumes, and distortions from different imaging techniques. Several groups have developed correlation methods using scripting software,^40–42^ and commercial software packages are also becoming available.^43^

Interfaces, such as grain boundaries, pose both challenges and opportunities for research with correlated methods. They can be represented in different spatial coordinates based on the resolution of the techniques used. For instance, determining the orientation of an indentation could be complicated if its plastic zone lies primarily within one grain or phase, while its elastic zone extends into a neighboring one. A common approach to address this is to analyze properties based on their distance from the interface. Tools for statistically examining these aspects could offer valuable insights into dislocation behavior, corrosion, grain-boundary segregation, and other interfacial phenomena.

The angle of incidence in various correlated techniques affects data registration. Techniques, such as rastered scanning electron microscopy (SEM) for EDS, which use normal incidence, create images with minor spherical aberrations over large areas, resulting in a regular sampling grid that minimizes distortions. If indentation deformation does not affect the technique (as with EDS), correlative imaging can be done post-indentation mapping, using the indentation grid for alignment.^37,44^ This cannot be done when the strain of the indentations alters the response of the correlated technique, such as Raman or EBSD. For the latter, similar plastic zone sizes to the beam interaction volume translate into inaccurate indexing of the grain’s orientation.

Oblique incidence techniques, such as the 70° sample tilt for EBSD, introduce correlation challenges due to perspective distortion from tilt angles, rotations of the region of interest, and surface topology variations. Accurate registration of features between data sets requires affine transformations. Additionally, the inclined probing volume varies with the sample’s atomic number and the electron beam’s voltage and current, highlighting the importance of considering discrete sampled volumes over the spatial grid.

Once these challenges are met, however, the power of these correlated microscopy techniques is immense. In samples with compositional gradients, the mechanical properties can be measured directly as a function of the composition using correlated indentation and EDS. This was first demonstrated on metallic meteorite samples^37^ to aid in deconvoluting the mechanical properties of the various phases. Correlated mapping techniques streamline the analysis of multiphase materials, materials degradation due to service environments, phase decomposition during heat treatments, and much more. However, one of the most powerful applications of EDS and nanoindentation mapping is for high-throughput investigation over combinatorial libraries with compositional gradients.^31,44^ This allows simultaneous investigation of entire sections of multicomponent phase diagrams using only a pair of correlated maps, providing more than a thousandfold increase in investigated combinations versus serial testing.

**Figure 4:**
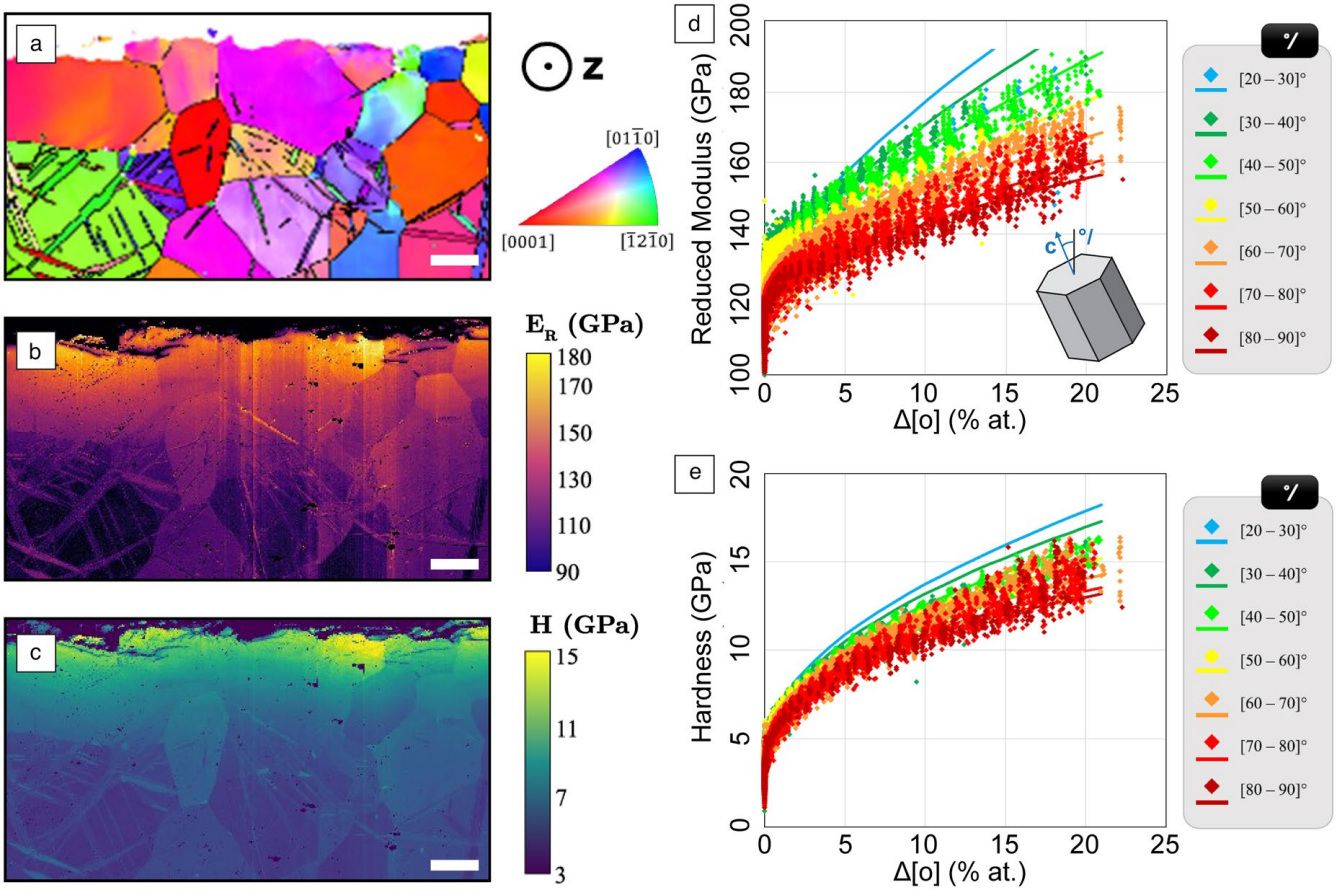
Correlated analysis of (a) crystal orientation from electron backscatter diffraction (EBSD) and mechanical properties (reduced modulus [b] and hardness [c]) from nanoindentation mapping of commercially pure (CP) titanium, allowing mechanical properties to be determined as a function of orientation and oxygen uptake (d and e) measured via electron probe microanalysis. (Scale bar = 25 μm). Reprinted with permission from Texier et al.^47^ © 2024 Elsevier.

Correlation with structural characterization techniques such as EBSD provides a further dimension of information: phase and crystallographic orientation. Phase information aids in data segmentation when composition or mechanical properties show significant overlap, while crystallographic orientation data are invaluable for characterizing mechanical anisotropy. This is particularly useful for deconvolving grain orientation effects from other factors, such as strain or oxygen concentration (**Figure** 4).

Significant progress has been made in anisotropy determination, particularly in elasticity. Modern indentation systems can precisely map the reduced elastic modulus of polycrystalline samples, enabling the extraction of elastic constants via the Vlassak–Nix model.^45^ This approach has been successfully demonstrated on cubic metals,^41,46^ and recently extended to hexagonal titanium alloys.^47^

Ongoing efforts aim to determine the relationships between plastic anisotropy in various crystal structures and their operative slip systems to assess the mechanical fingerprint of materials’ deformation behavior. A noteworthy example of this is shown in Figure 4, where correlated nanoindentation, electron probe analysis (EPMA), and EBSD were applied to study the effect of oxygen on the mechanical properties of titanium.^47^ Both EPMA and EBSD data are necessary so that the impact of anisotropy can be distinguished from the effect of oxygen. Combining approaches like this with testing combinatorial libraries enables high-throughput screening of thousands of alloy combinations in a single experiment.^48^

## Innovative approaches to data analysis

Precision, scale, and advanced data analysis must be balanced to handle large, high-velocity, and multimodal data sets. Three main challenges arise in deconvoluting the mechanical fingerprint of each phase within complex material domains:^36^ (1) environmental noise (e.g., vibrations, surface imperfections, temperature fluctuations), mainly affecting materials with subtle property gradients; (2) experimental artifacts (e.g., acquisition rate, tip wear, positioning errors); and (3) the intrinsic complexity of mechanical behavior, including elastic/plastic zone interactions, pileup, and strain-rate sensitivity.

Statistical methods have been foundational in nanoindentation mapping, notably in early cement paste studies,^33^ where they enabled phase identification and property extraction, typically through probability density function (PDF) and cumulative distribution functions (CDFs) deconvolutions. However, both methods struggle with complex, multimodal data sets and lack spatial context, highlighting the need for more advanced analysis techniques in the context of material innovation.

**Figure 5:**
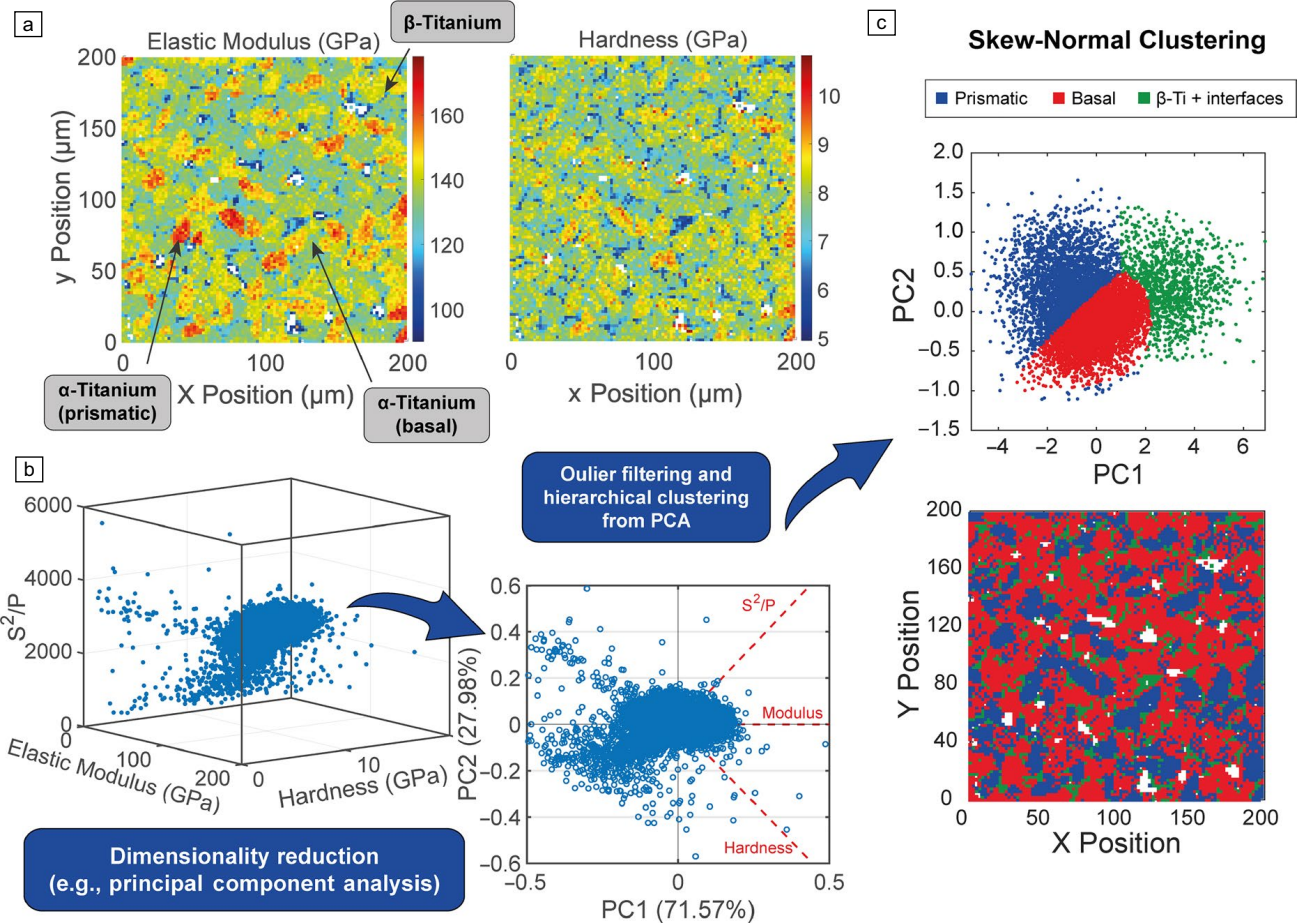
Possible workflow for phase deconvolution in high-speed maps using statistical machine learning. (a) Elastic modulus and hardness high-speed nanoindentation maps of a 3D printed α + β titanium alloy sample from Sergi et al.^50^ (b) Dimensionality reduction via principal component analysis (PCA). (c) Example of 2D innovative skew-normal clustering.

Given the availability of larger data sets, mechanical map analysis has evolved and allows multivariate approaches that account for correlations between measured properties (**Figure** 5a–b). Dimensionality reduction has become necessary. Principal component analysis (PCA)^49^ is commonly used to transform correlated variables—such as elastic modulus, hardness, and “stiffness squared over load”—into orthogonal components ranked by variance (Figure 5b). This process reduces dimensionality while preserving core mechanical behaviors and filtering out noise, such as outliers identified via Mahalanobis distance. This enables more effective identification of trends and a clearer analysis of material behavior.

After dimensionality reduction, innovative analysis approaches are now available and scaled with computational power. A key advancement is **statistical clustering** (retaining spatial information), mainly 2D Gaussian mixture model (GMM), which retains spatial information while capturing the shape and orientation of phases in feature space. However, its reliance on Gaussian assumptions can limit the accuracy of irregular structures, as discussed by Ortiz-Membrado et al. in WC-based cemented carbides.^29^ To address this, skew-normal distributions introduce flexibility (e.g., clustering in Figure 5c), improving phase boundary modeling, such as hard carbides in a softer Co matrix.

Parallel to statistically based approaches, machine learning (ML) techniques have expanded data analysis capabilities in mechanical microscopy. Broadly classified into supervised and unsupervised methods, initially unsupervised clustering methods such as k-means^51^ gained traction due to their scalability and ability to generate phase maps while retaining spatial information.^11,37,52^ However, its assumption of spherical clusters with equal variance can limit its effectiveness for data sets with irregular distributions or skewed features. More advanced alternatives, such as k-medoids,^53^ improve robustness by defining clusters based on actual datapoints (medoids) rather than centroids, reducing sensitivity to outliers.

For materials with hierarchical heterogeneity, such as multiphase metals or alloys, multistep clustering enhances phase identification. Hierarchical clustering methods such as agglomerative clustering can provide an initial broad categorization of data, which is then refined with methods such as GMM or k-medoids. Introducing temporary or fictitious clusters for outliers and interfacial regions improves accuracy, ensuring interfaces are analyzed rather than misclassified as noise or distorting primary clusters.

While 2D deconvolution and unsupervised clustering remain central to phase analysis, **supervised learning** is emerging as a powerful tool despite its reliance on manual feature engineering. By training on labeled data sets, supervised models map features to outputs, enabling precise classification and regression tasks. These methods are particularly effective when prior knowledge of phases or properties needs to be transferred to new data sets, such as in grain-boundary identification or correlative map registration. New possible approaches include Gaussian support vector machines (GSVM), k-nearest neighbors (kNN), and random forest (RF), requiring extensive data preprocessing, model training, and hyperparameter tuning. However, their high precision in phase classification makes them a valuable advancement in mechanical microscopy.^54–56^

The next leap is represented by deep learning, poised to transform mechanical microscopy by fully integrating indentation mapping with data science, shifting the mapping paradigm far from the oversimplifying modeling frameworks represented by physical interpretation models such as Oliver and Pharr.^3^ Rather than reducing data to spatial matrices of mechanical properties, modern deep learning approaches could leverage full load–displacement curves—and depth responses with CSM enabled—capturing rich information on “pop-in” events, which can indicate dislocation nucleation,^57,58^ avalanches and density changes,^59,60^ and often correlate with phase transformations, plasticity onset, cracking, and grain-boundary interactions.^60,61^ Localized features such as shear bands^62^ and surface irregularities such as roughness^60,63^ can also be inferred, along with strain bursts—higher magnitude discontinuities of load and/or displacement linked to twinning.^64^ Relaxation and creep responses provide insights into viscoelastic and time-dependent deformation behaviors,^65^ revealing rate-dependent mechanical properties. Furthermore, deviations from quadratic responses in fitting models could signal structural anisotropy, interface effects, transitions between elastic and plastic regimes, or the presence of subsurface particles and layered structures.^66^

Convolutional neural networks (CNNs) and recurrent neural networks (RNNs) address these challenges by recognizing morphological patterns and temporal dependencies in nanoindentation data. CNNs transform load-depth curves into image-like representations, enabling the detection of structural anomalies.^67^ RNNs, on the other hand, excel at capturing sequential trends in stiffness and creep behavior. Transfer learning further enhances these models by leveraging pretrained networks, reducing the need for large, annotated data sets, and broadening their applicability.

From this perspective on data analysis advancements, the optimal method depends on balancing accuracy, efficiency, and data set complexity, ensuring the best approach for phase identification and mechanical mapping in diverse materials.

## Future perspective (goals)

**Figure 6:**
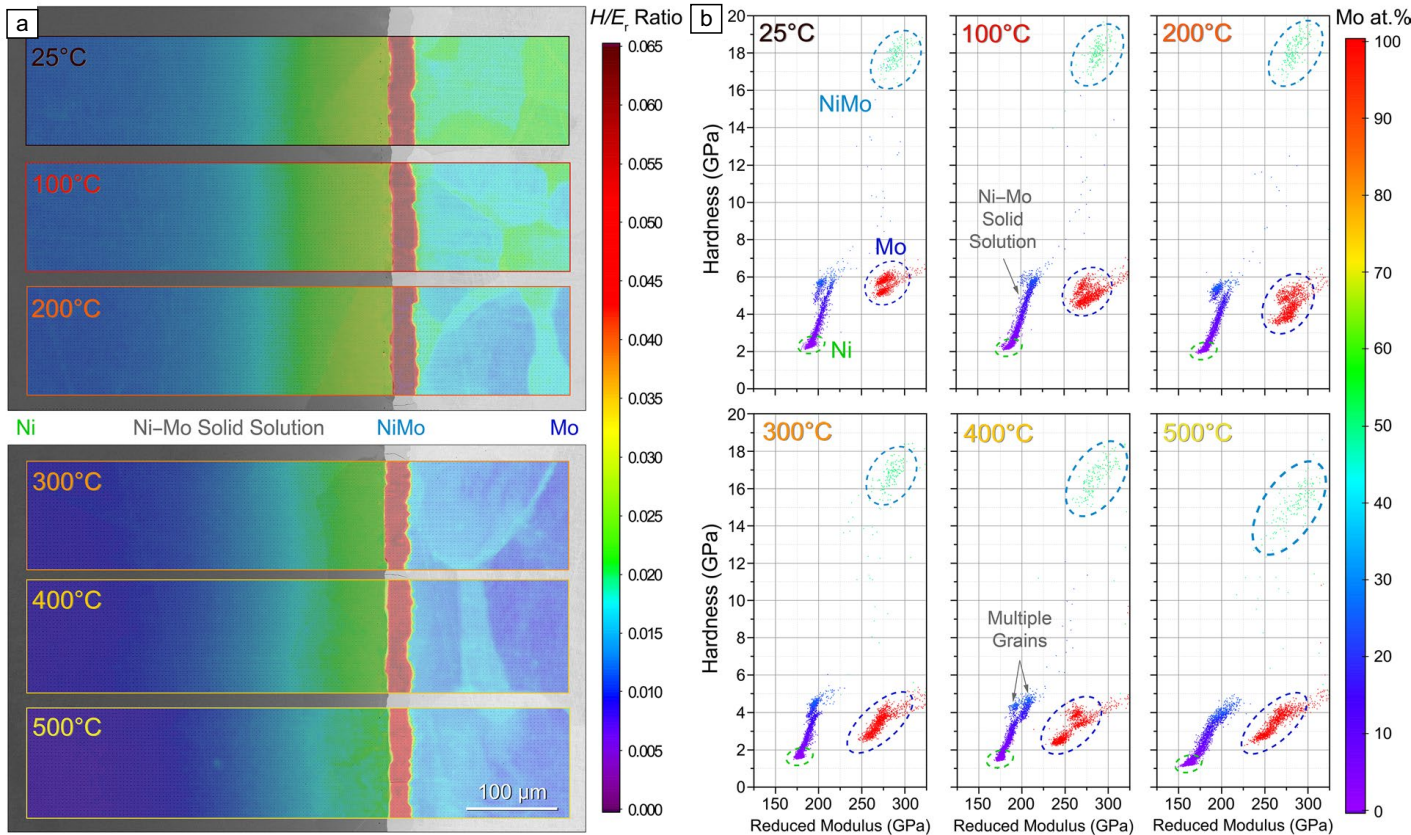
*Operando,* correlative, and combinatorial mechanical microscopy of a Ni–Mo diffusion couple. (a) *H/E*_r_ ratio maps overlaid on backscattered electron micrographs of adjacent regions of the Ni–Mo diffusion junction acquired at temperatures from 25 to 500°C. (b) Mechanical property distributions with datapoints color-coded using correlated energy-dispersive x-ray spectroscopy to illustrate compositional gradients and primary phases. Figure courtesy of Oxford Instruments—FemtoTools AG.

As control electronics continue to improve, near real-time mechanical mapping at high resolutions is becoming feasible. Correlation methods with other analytical mapping techniques are likely still in their early days of development. They are expected to find mainstream applications within many fields of science, not only materials science. Additional system developments will enable *operando* mechanical mapping across various temperatures and environmental conditions, completing the materials science tetrahedron of structure, processing, properties, and performance by evaluating materials in service conditions at the microstructural level.** Figure** 6 shows an example of this, where high-temperature nanoindentation mapping was recently employed with correlated EDS in a combinatorial manner over a Ni–Mo diffusion couple. This allows the simultaneous visualization of the temperature dependence of the mechanical properties of a wide range of compositions and phases in a single graph.

Moreover, automating multimodal integration and enhancing the precision of mechanical, structural (EBSD), and compositional (EDS) correlations will enable more systematic analyses, such as linking crystallographic orientation with mechanical behavior or using compositional gradients to refine phase boundary and diffusion profile characterization.

On the real-time nanoindentation mapping side, it must extend beyond the mere data analysis, integrating ML-driven adaptive decision-making to optimize instrumentation and enable intelligent, real-time mapping adjustments—dynamically optimizing spatial resolution and sampling density based on microstructural predictions (e.g., gradient-based regressions and clustering of data set sub-instances for adaptive data densities), minimizing experimental time—and * in situ* monitoring of measurement quality; indeed, indenter wear depends on load, depth, material hardness, and chemical affinity, with minimal wear after 10,000 indentations on sapphire,^68^ but rapidly degrading diamond tips when testing at higher temperatures, especially on ferrous metals.^69^ Deep learning automated tip-wear analysis becomes crucial.^70^

The backbone of these advancements is ultimately rooted in the establishment of open-source and findable, accessible, interoperable, reusable (FAIR) databases and ontologies for indentation data.

In the long term, nanoindentation will become a fully automated platform for correlative microstructural analysis, integrating ML, and multimodal data sets.

## Summary

In the previous sections, we analyzed the innovations in hardware, data visualization, data analysis, and application fields that have enabled nanoindentation mapping to become a powerful tool for mechanical microscopy of microstructures.

High-speed nanoindentation presents several challenges, especially when integrating mechanical maps with structural and compositional data from methods such as EBSD and EDS. Challenges such as spatial misalignment, map registration and correlation, noise, and varying resolutions necessitate advanced data alignment techniques, including affine transformations, to maintain accuracy. Additionally, the rapid indentation process can (1) generate strain rates of up to 3/s, triggering strain rate issues, and (2) potentially introduce plasticity errors linked to rapid CSM oscillations. The correct microstructural investigation that avoids misleading interpretations is primarily based on the selection (or absence) of data interpolation techniques, ranging from nearest neighbor to bicubic methods. Each has tradeoffs between resolution and potential data distortion.

Second, on the data analysis advancements. In this work, we discussed the role of statistical deconvolution methods such as GMM and ML techniques, including k-means clustering, to discern patterns in complex, high-dimensional data. Deep learning is the crucial leap forward for advanced complex mechanical data extraction, albeit at the cost of meticulous data annotation.

Current research is centered on *operando* testing in variable environments and the creation of adaptive algorithms for real-time data refinement. These efforts are expected to enhance applications in material design and structural optimization.

## Data Availability

This article is primarily a review and is based on previously published literature, which is appropriately cited throughout the text. Any original data generated during this study are available from the corresponding author upon reasonable request.
